# A temperature model for laser lithotripsy

**DOI:** 10.1007/s00345-020-03357-y

**Published:** 2020-07-24

**Authors:** J. G. Williams, L. Goldsmith, D. E. Moulton, S. L. Waters, B. W. Turney

**Affiliations:** 1 Mathematical Institute, Andrew Wiles Building, Woodstock Road, Oxford, OX2 6GG UK; 2 Nuffield Department of Surgical Sciences, John Radcliffe Hospital, University of Oxford, Headington, Oxford, OX2 6GG UK

**Keywords:** Lithotripsy, Mathematical modelling, In vitro experiments, Thermal tissue damage

## Abstract

**Objective:**

To derive and validate a mathematical model to predict laser-induced temperature changes in a kidney during kidney stone treatment.

**Methods:**

A simplified mathematical model to predict temperature change in the kidney for any given renal volume, irrigation flow rate, irrigation fluid temperature, and laser power was derived. We validated our model with matched in vitro experiments.

**Results:**

Excellent agreement between the mathematical model predictions and laboratory data was obtained.

**Conclusion:**

The model obviates the need for repeated experimental validation. The model predicts scenarios where risk of renal tissue damage is high. With real-time knowledge of flow rate, irrigating fluid temperature and laser usage, safety warning levels could be predicted. Meanwhile, clinicians should be aware of the potential risk from thermal injury and take measures to reduce the risk, such as using room temperature irrigation fluid and judicious laser use.

## Introduction

Over the last 30 years, Holmium lasers have been used to fragment stones within the urinary tract. Initially, low-power lasers (20 Watts) were used to fragment the stones into pieces for manual basket extraction. Relatively few high-energy impulses were delivered. Over time, lasers for stone surgery have become more powerful (up to 120 Watts) to provide the ability to modulate the frequency and pulse energy across a greater range. This has made the technique of “dusting” feasible. In this technique, the laser is used at a higher frequency with a lower energy to gradually break off tiny pieces of stone that can be passed spontaneously in the urine and do not require time-consuming extraction. Other surgical techniques such as “pop-corning” rely on firing the laser at higher power—typically around 40 Watts—for a prolonged period of time to agitate the stone fragments in the kidney. As the fragments move around, they fragment into smaller pieces when they come in contact with the laser. In the last year, the new thulium-doped fibre laser has been launched for clinical use and offers the potential for delivering even higher power levels. As lasers have evolved, the power (energy/second) delivered can be increased. An unintended consequence is the risk of thermal tissue damage due to heating of the irrigation fluid within the ureter or renal pelvis.

The potential for thermal tissue damage during holmium laser lithotripsy, and its dependence on procedural parameters, is currently under investigation. Recent in vitro [1, 2] and in vivo [3] experimental work has aimed to measure fluid temperatures resulting from holmium laser activation. These studies considered different laser settings—energy and pulse frequency—as well as *irrigation* flow rate; irrigation is the continuous delivery of saline solution to clear stone fragments resulting from lithotripsy [1–3]. All measured high fluid temperatures—particularly with high laser power and low irrigation flow—in their experimental setups and voiced a concern for thermal tissue damage as indicated by the commonly used $t_{43}$ metric [4].

Mathematical modelling provides a platform for predicting temperatures within the renal pelvis, and subsequent risk of thermal tissue damage, without the need to experimentally test every clinical condition. A computational model to predict the distribution of temperature over time and space due to holmium laser lithotripsy was developed in a commercial finite-element software, COMSOL Multiphysics, by [3]. The model includes equations for heat transfer through the fluid, the solid boundary, and couples temperature to fluid flow through temperature-dependent fluid properties. Simulations were performed to mimic their in vivo and in vitro experimental setups, and the calculated volume-averaged temperatures were in good agreement with their experimental results. However, implementation of this model requires some knowledge of COMSOL Multiphysics, and a simulation for a single set of parameter values requires a few minutes of computational time.

Therefore, it is of interest to investigate whether a significantly reduced mathematical model can still capture the necessary physics to provide an accurate estimate of volume-averaged fluid temperature as a function of relevant clinical parameters. To motivate our model, we conduct a series of in vitro experiments, similar to those performed by [1], although with different irrigation fluid temperatures and a larger experimental vessel. This provides two sets of experimental data against which to validate our mathematical model. The model itself comprises a single analytical expression, which can be evaluated with no computational expense for any clinical parameters on a standard calculator. We determine good agreement between our model and experimental data, along with the experimental data of [1]. We then discuss the potential for thermal tissue damage as predicted by the validated mathematical model, and predict clinically safe ranges of laser settings and irrigation flow rate such that fluid temperatures remain below a critical threshold.

## Experimental setup

**Fig. 1:**
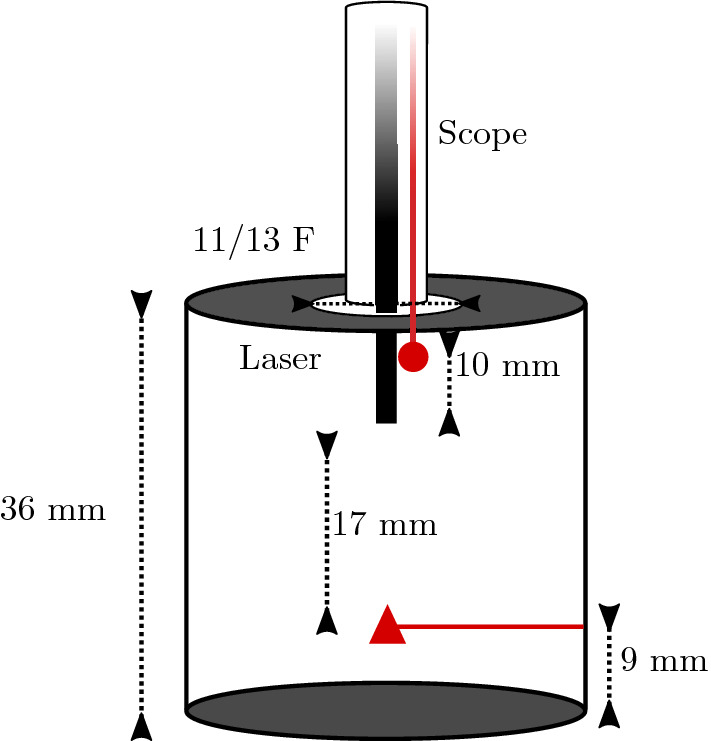
The laser fibre is 10 mm distal to the scope tip. Two thermocouples measured temperature over time positioned at the scope tip and 9 mm from the base of the container. These are indicated by a circle and triangle, respectively. Diagram not to scale

Wet-lab experiments consisted of a 38.3-mL cylindrical vessel, submerged in a 1-L container, both filled with room temperature saline. A Boston Scientific LithoVue™ ureteroscope was positioned with its tip flush with an 11/13 F hole at the top of the container. A 365-μm laser fibre (Flexiva™  Boston Scientific) was inserted through the ureteroscope and secured 10 mm distal to the ureteroscope end (Fig. 1). Two T-type theromocouple wires were positioned proximal and distal to the laser (red circle and triangle, respectively, in Fig. 1) and measured temperature at 0.1 s intervals. Irrigation was switched as we commenced temperature recording. After 20 s, the laser was switched on for a total of 60 s. After the laser was switched off, irrigation was maintained and temperatures recorded for a further 20 s.

We considered six sets of experimental settings—two laser powers and three flow rates—summarised in Table 1. We performed five runs of each configuration.

**Table 1:** Summary of experiment settings

Experiment no.	Laser setting	Flow rate	No. runs
1	$1\text { J}\times 40\text { Hz} = 40\text { Watts}$	0 mL min$^{-1}$	5
2	$1\text { J}\times 40\text { Hz} = 40\text { Watts}$	20 mL min$^{-1}$	5
3	$1\text { J}\times 40\text { Hz} = 40\text { Watts}$	40 mL min$^{-1}$	5
4	$0.5\text { J}\times 20\text { Hz} = 10\text { Watts}$	0 mL min$^{-1}$	5
5	$0.5\text { J}\times 20\text { Hz} = 10\text { Watts}$	20 mL min$^{-1}$	5
6	$0.5\text { J}\times 20\text { Hz} = 10\text { Watts}$	40 mL min$^{-1}$	5

Our experiments were similar to those performed by [1], although with different values for the vessel volume and material properties, initial fluid temperature, and irrigation temperature. The volume of our experimental vessel (38.3 mL) is representative of a typical kidney, whereas the volume of the experimental vessel used by [1] is more indicative of an isolated calyx (5.9 mL). We will consider both sets of experiments in this article to compare to our modelling predictions. We refer to experiments performed by [1] as Set A and our experiments as Set B. All experiment parameters are summarised in Table 2. Adjusting for the position of the laser fibre with respect to the scope had no notable effect on our findings (see Appendix B).

**Table 2:** Experimental parameters

Quantity	Symbol	Value	Unit
Thermal conductivity (water, $25\,^{\circ }\text {C}$)	*k*	$6.06\times 10^{-1}$	W m$^{-1}$ K$^{-1}$
Density	$\rho$	$1.00\times 10^3$	kg m$^{-3}$
Specific heat capacity (water)	*c*	$4.18\times 10^3$	J K$^{-1}$ kg$^{-1}$
Experiment parameters in [1] (Set A)			
Test tube length	–	$7.50\times 10^{-2}$	m
Test tube diameter	–	$1.00\times 10^{-2}$	m
Container volume	*V*	$5.89\times 10^{-6}$	m$^3$
Water bath temperature	$T_0$	37	$\,^{\circ }\text {C}$
Irrigation temperature	$T_{\text {in}}$	23	$\,^{\circ }\text {C}$
Firing time	$t_{\text {f}}$	60	s
Our experiment parameters (Set B)			
Container length	–	$3.60\times 10^{-2}$	m
Container diameter	–	$3.68\times 10^{-2}$	m
Container volume	*V*	$3.83\times 10^{-5}$	m$^3$
Water bath temperature	$T_0$	22	$\,^{\circ }\text {C}$
Irrigation temperature	$T_{\text {in}}$	22	$\,^{\circ }\text {C}$
Firing time	$t_{\text {f}}$	60	s

## Theoretical model

A mathematical model for spatially averaged temperature as a function of time *T*(*t*), in a fluid-filled vessel of volume *V*, subject to irrigation at flow-rate *Q* and temperature $T_{\text {in}}$ and laser lithotripsy at power *W*, is derived in Appendix C. The model comprises an analytic formula predicting an exponential temperature rise during lasering, followed by an exponential decay when the laser is switched off. If the laser is switched on for sufficient time, a steady-state temperature is reached. The volume of the vessel sets only the time required to reach the steady-state temperature, and not its magnitude. The model takes, as input parameters, *V*, *Q*, *W*, $T_{\text {in}}$, along with the initial temperature in the vessel $T(0) = T_0$, the time the laser is switched on $t_1$, the time the laser is switched off $t_2$, and *k*, $\rho$, and *c* which are the thermal conductivity, density, and specific heat capacity of the irrigation fluid, respectively.

The final model parameter is $\beta = hs$, where *h* is the heat transfer coefficient through the walls of the vessel and *s* is the cross-sectional area of the walls through which heat diffuses. As *h* is highly dependent on the material properties of the vessel under consideration, we treat this as an unknown parameter, and determine it through a fit to experimental data. The material properties of the vessel in Set A and Set B are potentially different, so we determine one value of $\beta$ for each data set.

We define the laser firing time as $t_{\text {f}} = t_2 -t_1$ and the temperature change as $\Delta T(t) = T(t) - T_0$.

### Fit for $\beta$

**Fig. 2:**
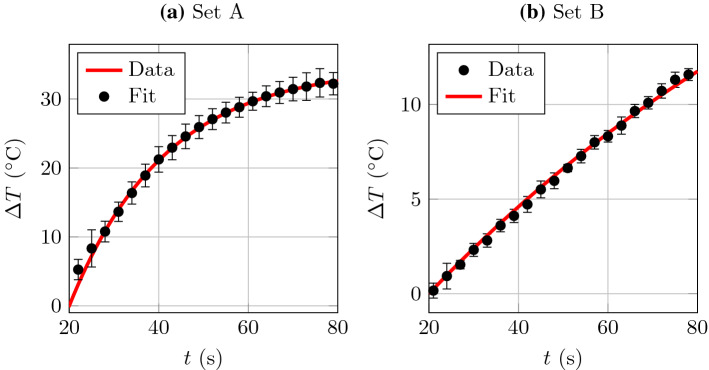
The unknown parameter was obtained by fitting the analytical solution for $Q = 0$ mL/min, ${\mathcal {W}} = 40$ Watts:** a** data from Set A,** b** data from Set B. The best-fit values are** a** $\beta \approx 1.14$ Watts/$^{\circ }\text {C}$ and** b** $\beta \approx 1.36$ Watts/$^{\circ }\text {C}$

We fit for $\beta$, which characterises the thermal conductivity through the walls of the experimental vessel. For both sets of experiments A and B, we fit for $\beta$ using the case where $Q = 0$ mL min$^{-1}$ and the laser is switched on with power $W = 40$ Watts. The experimental data, along with the lines of best fit, are plotted in Fig. 2a, b for Set A and Set B, respectively.

The best-fit values of $\beta$ are found to be

1 $$\begin{aligned} \beta _{\text {A}}\approx & {} 1.15\text { Watts/}^{\circ }\text {C}, \nonumber \\ \beta _{\text {B}}\approx & {} 1.36\text { Watts/}^{\circ }\text {C}, \end{aligned}$$

for the experiments from Set A and Set B, respectively.

## Results

Using the best-fit values for $\beta$, Eq. (1), we have the required information to compare the experimental data from both sets to the predictions of the theoretical model.

### Comparison with experimental data

**Fig. 3:**
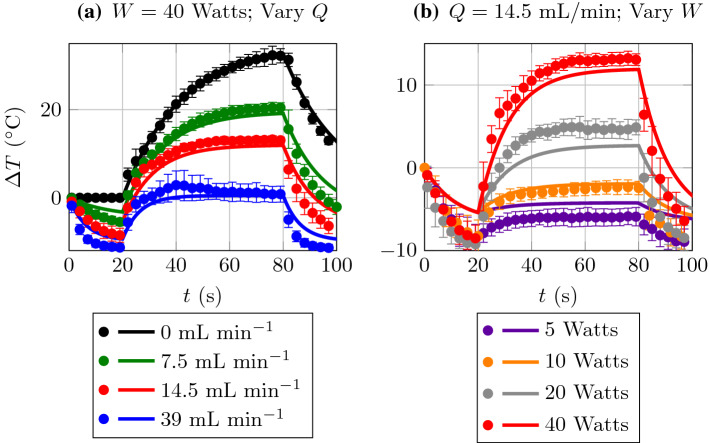
A comparison of the model predictions (solid lines) with the experimental data from Set A (symbols)

In Fig. 3, we present the data extracted from Set A (symbols) along with model predictions (solid lines). Experimental parameters used in the model are given in Table 2.

In Fig. 3a, we consider fixed $W = 40$ Watts, and vary *Q*. We note that although $\beta$ was only obtained through a fit to the exponential rise for $Q = 0$ mL min$^{-1}$ it accurately predicts temperatures for $Q = 7.5$, 14.5, and 39 mL min$^{-1}$. In Fig. 3b, we fix $Q = 14.5$ mL min$^{-1}$ and vary laser power *W*. The data and model prediction for $W = 40$ are repeated from the red data set and model prediction in Fig. 3a. Again, we observe good agreement between theory and experiment. We speculate that discrepancies between theory and data in Fig. 3b may be due to variability in flow rates which are given in [1] as ranges: $Q = 0$, $7-8$, $14-15$, $38-40$ mL min$^{-1}$—estimated from a measured driving pressure head.

**Fig. 4:**
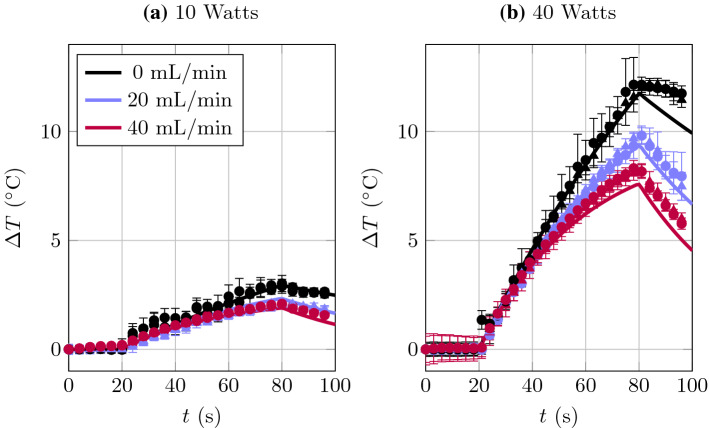
A dimensional comparison of the model predictions (solid lines) with the experimental data (symbols). Triangles are from the thermocouple 9 mm from the base of the container and circles from the thermocouple at the level of the scope tip (see Fig. 1)

In Fig. 4, we present experimental results from Set B (symbols) compared to the predictions of the mathematical model (solid lines). Experimental parameters are given in Table 2. Figure 4a is for $W = 10$ Watts and Fig. 4b is for $W = 40$ Watts. The triangle and circle symbols in Fig. 4 indicate the two different temperature probes with corresponding symbols in Fig. 1. We observe good agreement between theory and experiment in both setups. The overlapping triangle and circle data points in Fig. 4 validates the use of a spatially averaged mathematical model by indicating a fairly homogeneous temperature throughout the experimental vessel.

It is illuminating to compare Figs. 3a to  4b, which are both for $W = 40$ Watts, and observe the comparatively larger temperature increases observed in the experimental data of Set A. This is due to the volume disparity between the two experiment sets. The mathematical model described in Appendix C predicts that the time required to reach the steady-state temperature is inversely proportional to the volume of the vessel. Thus, within a smaller vessel, the temperature equilibrates more quickly, and thus higher temperatures are achieved for the same firing time than in a larger vessel. The inverse relationship between rise time and vessel volume is captured by the shallower slopes of the temperature curves in Fig. 4 when compared to the curves in Fig. 3.

### Effects of volume, flow rate, and laser power

**Fig. 5:**
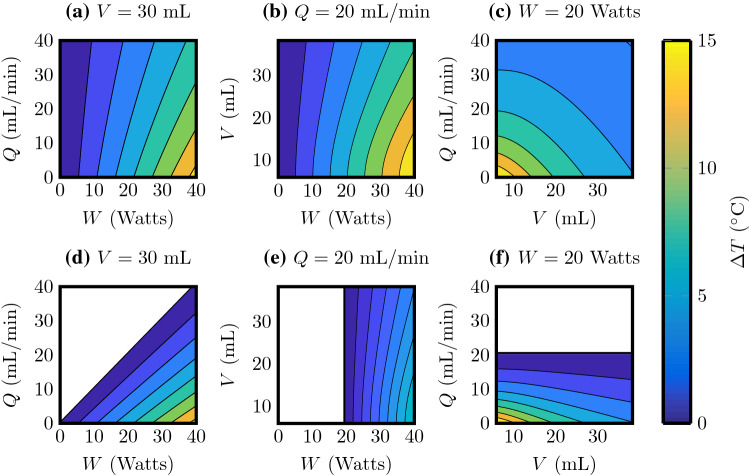
Predicted temperature change after 60 s of laser firing for $T_0 = 37\,^{\circ }\text {C}$. In the top row $T_{\text {in}} = 37\,^{\circ }\text {C}$ and in the bottom row $T_{\text {in}} = 23\,^{\circ }\text {C}$. The colours provide $\Delta T$ and white regions indicate where $\Delta T < 0$.** a**,** d** $V = 30$ mL and *Q*, *W* varied.** b**,** e** $Q = 20$ mL min$^{-1}$ and *V*, *W* varied.** c**,** f** $W = 20$ Watts and *Q*, *V* varied

The separate effects of volume *V*, flow rate *Q* and laser power *W*, are illustrated as contour plots in Fig. 5 after firing for $t_{\text {f}} = 60$ s.

For all plots in Fig. 5 the initial temperature $T_0 = 37\,^{\circ }\text {C}$. The top row of plots in Fig. 5 are for $T_{\text {in}} = 37\,^{\circ }\text {C}$ and the bottom row for $T_{\text {in}} = 23\,^{\circ }\text {C}$. Colours denote temperature change $\Delta T$, and white regions of the plots present for $T_{\text {in}} = 23\,^{\circ }\text {C}$ (bottom row) indicate where $\Delta T < 0$. We note an increase in $\Delta T$ with *W*, a decrease in $\Delta T$ with *Q*, and a decrease in $\Delta T$ with *V*. Figure 5 also illustrates, by comparing the bottom row with the top row, the advantage of irrigating at room temperature ($T_{\mathrm{in}}= 23\,^{\circ }\text {C}$) rather than body temperature ($T_{\mathrm{in}}= 37\,^{\circ }\text {C}$) to maintain low temperatures.

### Thermal dose

Sustained temperatures above 43 °C are known to cause thermal damage to many biological tissues [4].

**Fig. 6:**
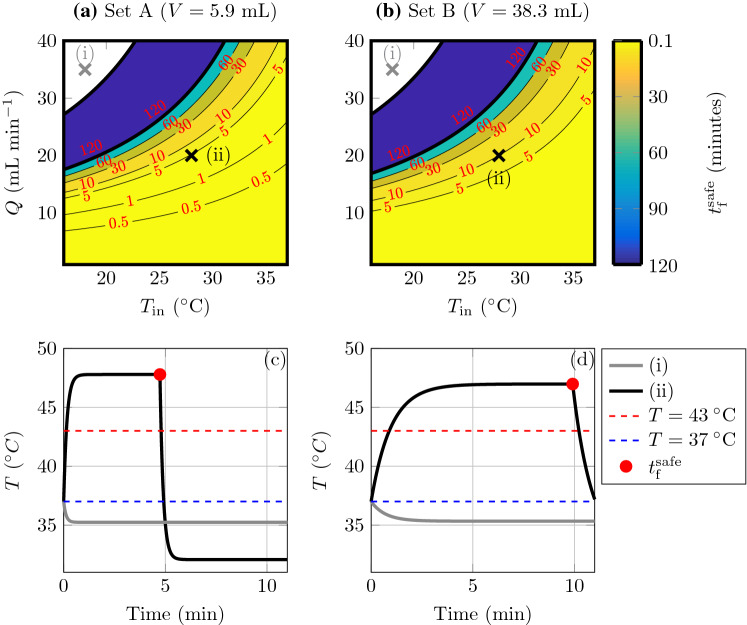
**a** and** b** Predicted $t_{\text {f}}^{\mathrm{safe}}$ (in minutes), as a function of $T_{\text {in}}$ and *Q*, such that $t_{43}=120$ min for** a** conditions for experiment Set A and** b** conditions for experiment Set B.** c** and** d** Example temperature curves for labelled points (i) (gray) and (ii) (black) in (**a**) and (**b**), respectively. Red dots indicates $t_{\text {f}}^{\mathrm{safe}}$, the dashed red line shows $T = 43\,^{\circ }\text {C}$, and the dashed blue line shows $T = 37\,^{\circ }\text {C}$. In all figures, $T_0 = 37\,^{\circ }\text {C}$ and $W = 40$ Watts. The two thicker black lines in (**a **) and (**b **) denote $T^{\star } = 37\,^{\circ }\text {C}$ and $T^{\star } = 43\,^{\circ }\text {C}$

A common metric for evaluating thermal dose is to convert a given temperature curve to the equivalent time at constant $43\,^{\circ }\text {C}$ by the formula

2 $$\begin{aligned} t_{43} = \int R^{43 - T(t)}\mathrm{d}t, \end{aligned}$$

where

3 $$\begin{aligned} R = {\left\{ \begin{array}{ll} 0, \quad &{}t \le 37\,^{\circ }\text {C}, \\ 0.25, \quad &{}37<t < 43\,^{\circ }\text {C}, \\ 0.5, \quad &{}t \ge 43\,^{\circ }\text {C}. \end{array}\right. } \end{aligned}$$

Equation (2) indicates that if $T(t) \equiv 43\,^{\circ }\text {C}$ then $t_{43} = t$. Any increase in *T* above $43\,^{\circ }\text {C}$ results in an exponential increase in the thermal dose. This mathematical description of thermal dose is based on evidence from in vitro and in vivo systems [4]. The time threshold for damage to occur is often taken to be $t_{43} = 120$ min [3].

Using our mathematical model for temperature as a function of system parameters, we can determine the firing time $t_{\mathrm{f}}$ such that $t_{43}$ remains below the safe thermal dose threshold of 120 min. We will refer to this as $t_{\mathrm{f}}^{\text {safe}}$. Details of the numerical procedure to calculate $t_{\text {f}}^{\mathrm{safe}}$ are provided in Appendix D. In Fig. 6a, b, we display contour plots of $t_{\text {f}}^{\mathrm{safe}}$ as a function of $T_{\mathrm{in}}$ and *Q* for the experimental conditions for Set A and Set B, respectively. The white region indicates where $T^{\star } \le 37\,^{\circ }\text {C}$, and hence, by Eqs. (2), (3), $t_{43}\equiv 0$, and thus $t_{\text {f}}^{\mathrm{safe}}= \infty$. Example curves from this region for $T_{\mathrm{in}}= 18\,^{\circ }\text {C}$, $Q = 35$ mL min$^{-1}$ are shown in gray in Fig. 6c, d, where it can be seen that the curves plateaus below $T = 37\,^{\circ }\text {C}$, indicated by the dashed blue line. As $T^{\star }$ is independent of *V*, the white region is nearly identical between Fig. 6a, b (any discrepancies due to the slightly different value for $\beta$ used for the two experiment setups). A second set of example curves are shown in Fig. 6c, d for $T_{\mathrm{in}}= 28\,^{\circ }\text {C}$ and $Q = 20$ mL min$^{-1}$. The value of $t_{\text {f}}^{\mathrm{safe}}$ is indicated by the red dots. We see, by comparing the black curve in Fig. 6c to the black curve in Fig. 6d, the comparatively sharper temperature rise due to the smaller volume. Thus, $t_{\text {f}}^{\mathrm{safe}}$ for Set A conditions is less than $t_{\text {f}}^{\mathrm{safe}}$ for Set B conditions if $T^{\star } > 37\,^{\circ }\text {C}$.

## Discussion

We conducted a set of in vitro experiments to measure fluid temperature over time as a result of holmium laser lithotripsy. Resulting temperature curves agreed qualitatively with [1, 3] and [2], with temperatures rising after the initiation of laser activation to reach a stable, elevated value, before decreasing after the laser was switched-off. We subsequently derived a mathematical model from conservation of energy principles, neglecting spatial temperature variation and considering only the volume-averaged temperature over time. This produced a single equation with which to compare against experimental data, and we fit for the parameter representing the thermal conductivity through the walls of the experimental vessel. We obtained excellent quantitative agreement with our experimental data and the data from [1] for all laser settings and irrigation flow rates considered.

The model predicts an increase in fluid temperature within the kidney with laser power and a decrease with irrigation flow rate. Irrigation flow rate may be altered clinically by either the adjusting the inflow (e.g. increasing pressure on the irrigation fluid) or outflow (e.g. use of access sheath) from the kidney. Our results corroborate the key findings of [1–3]. We also determined the volume of the working space as a key parameter in controlling the temperatures achieved; a smaller volume reaches higher temperatures more quickly, although the equilibrium temperature, which will be reached if the laser is fired for sufficient time, is independent of volume. This raises a pertinent question: *what is the relevant volume of the collecting system during laser lithotripsy?* Large variation in pelvicalyceal volume are reported in the literature [5], and of course, the comprising features—the renal pelvis and multiple calyces—have different sizes. Therefore, patient-specific anatomy, as well as the location of a stone, may have a significant effect on fluid temperature and the potential for thermal damage.

In addition to irrigation flow rate—or equivalently driving pressure—the temperature of irrigation fluid before it enters the patient also affects renal temperatures during laser lithotripsy. As relatively small quantities of fluid are used for irrigation, there will only be a minimal effect on global body temperature. However, local heating of irrigation fluid by higher powered laser use has a risk of detrimental effect on renal tissue. Due to the specific heat capacity of water, clinicians should be advised to use room temperature fluids (rather than warmed fluids) for irrigation to minimise thermal damage to the kidney from laser use.

In our experiments, we recorded temperature at two locations within the fluid-filled cylinder, and found no significant difference between the readings. However, we realise that our simplified cylindrical geometry does not represent the complex anatomical structure of the renal collecting system, and that changes in geometry may lead to spatially heterogeneous temperatures. Thus, although our mathematical model has the benefit of computational simplicity, it only predicts a single volume-averaged temperature. This will always be a lower bound on the maximum temperature within the kidney and thus, model results must be applied with caution to not overlook potential hot spots proximal to the laser fibre. More intensive simulations, such as those performed by [3], are required to predict the spatial distribution of temperature. Another limitation of the mathematical model is the need to determine the thermal conductivity of the material surrounding the fluid-filled container experimentally; from an in vivo standpoint, this will be the material properties of the renal tissue. Thus, further characterisation of renal volume and tissue properties from in vivo experimental data will both contribute to improving the accuracy of our mathematical model.

It is also important to note that in our experiments and mathematical model, we consider a minute of uninterrupted lasering. However, it is unusual for a laser to be used continuously for long periods of time during a case due to practical factors such as repositioning of the laser fibre, movement of the stone and inadequate view of the stone. Further work to investigate how typical “operator duty cycle” impacts on temperature changes. This is within the scope of the model provided the on/off intervals and laser settings are known.

## Conclusion

This validated mathematical model allows prediction of the change in temperature within a kidney for any given renal volume, irrigation flow rate, irrigation fluid temperature, and laser power. The model obviates the need for repeated experimental validation. The model predicts scenarios where risk of renal tissue damage are high. With real-time knowledge of flow rate, irrigating fluid temperature and laser usage, safety warning levels could be predicted. In the meanwhile, clinicians should be aware of the potential risk from thermal injury and take measures to reduce the risk such as using room temperature irrigation fluid and judicious laser use.

## Figures and Tables

**Fig. 7:**
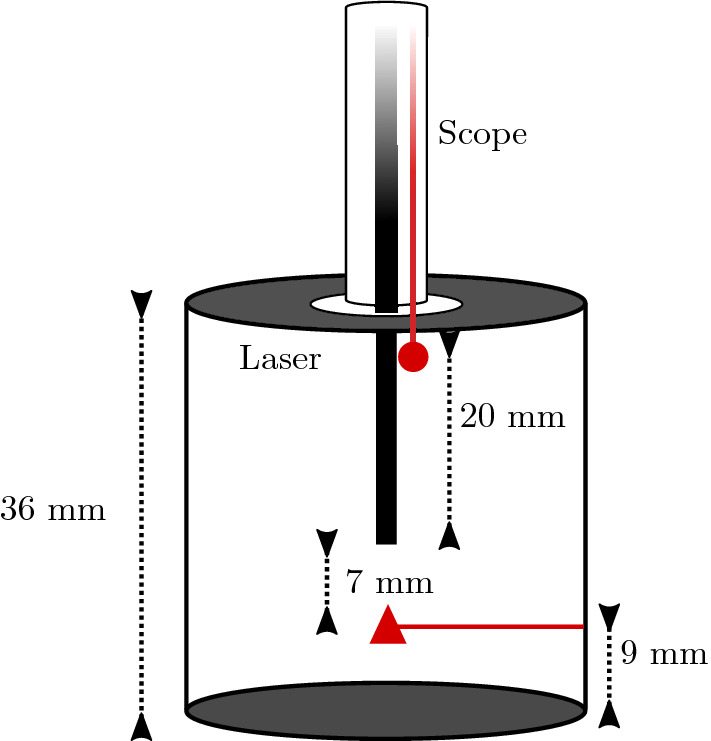
The laser fibre is 20 mm distal to the scope tip. Two thermocouples measured temperature over time positioned at the scope tip and 9 mm from the base of the container. These are indicated by a circle and triangle, respectively. Diagram not to scale

**Fig. 8:**
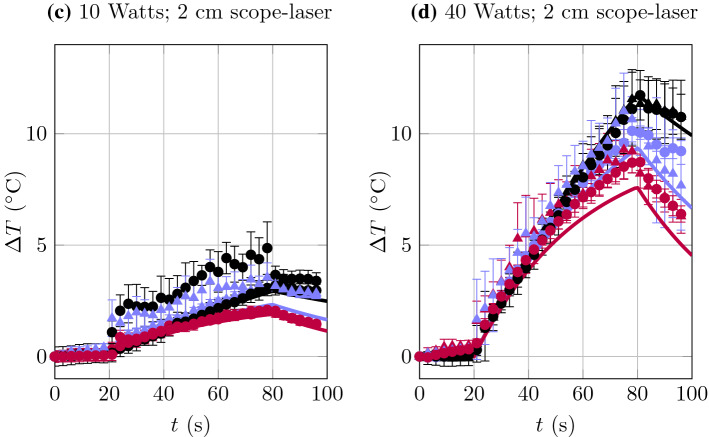
A dimensional comparison of the model predictions (solid lines) with the experimental data (symbols). Triangles are from the thermocouple 9 mm from the base of the container and circles from the thermocouple at the level of the scope tip (see Fig. 7)

**Fig. 9:**
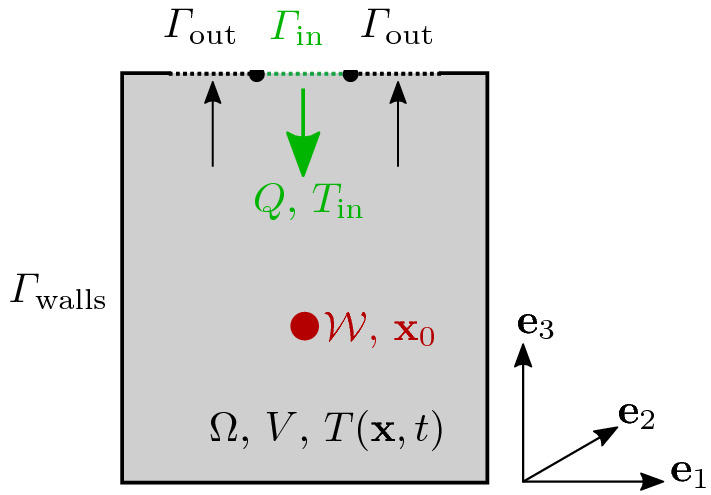
The considered domain

## Appendix A: Data analysis

The raw data from the experiments were obtained as temperature measurements ($^{\circ }$C) at 0.1-s intervals from each thermocouple. The temperature difference, $\Delta T$, was extracted from the data by subtracting the initial temperature reading.

### Error estimation

The thermocouples had a readability of $10^{-3}\,^{\circ }\text {C}$. Therefore, the error in the temperature measurements were taken to be $\delta = 5\times 10^{-4}\,^{\circ }\text {C}$. The error for the temperature difference, $\Delta T$, is hence $\sqrt{2}\delta$. We assume that the standard deviation of the measured temperatures over each set of five runs and the measurement errors due to the readability of the thermocouples are independent and thus our total error estimation is

4 $$\begin{aligned} T.E. = \sqrt{\sigma ^2 + 2\delta ^2}, \end{aligned}$$

which is taken as the vertical error bars on the experimental data in Figs. 4 and 8.

## Appendix B: Laser positioned 2 cm distal to scope tip

We also considered the experimental setup pictured in Fig. 7, where the laser is positioned 20 mm (as opposed to 10 mm) distal to the scope tip. The experimental settings were the same as those summarised in Table 1.

Experimental results are plotted against model predictions in Fig. 8. The agreement is good between theory and experiment, although there is more variability in the triangle data points in Fig. 8; these correspond near the base of the vessel, positioned only 7 mm from the laser fibre (Fig. 7). Thus, a potential hypothesis for this variability is laser-thermocouple interference.

## Appendix C: Theoretical formulation

We assume a three-dimensional kidney calyx of arbitrary geometry, $\Omega$ (Fig. 9). The temperature in the cavity is $T({\mathbf {x}},t)$, a function of spatial coordinate ${\textit{\textbf{x}}}\in \Omega$ and time, *t*.

Irrigation fluid, with pointwise velocity ${\mathbf {u}}({\mathbf {x}},t)$ enters the calyx at a temperature, $T_{\text {in}}$ (which is typically taken to be body temperature, $37\,^{\circ }\text {C}$, but can also be room temperature, $21-23\,^{\circ }\text {C}$). There is a net flux of irrigation fluid fluid, *Q*, through the cavity. We assume that the laser inputs power density as a function of time, ${\mathcal {W}}(t)$ (units Watts/m$^3$) at a fixed point, ${\mathbf {x}}_0$. Conservation of energy provides

5 $$\begin{aligned} \rho c\frac{\partial T}{\partial t} + \nabla \cdot {\mathbf {J}}= & {} {\mathcal {W}}(t)\delta ({\mathbf {x}}-{\mathbf {x}}_0), \nonumber \\ {\mathbf {J}}= & {} \rho c {\mathbf {u}}T - k \nabla T, \end{aligned}$$

where $\rho$, *c*, and *k* are the density, specific heat capacity, and thermal conductivity of the irrigation fluid, respectively, and ${\mathbf {J}}$ is the heat flux. We assume heat is advected (without diffusing) in and out of the calyx,

6a $$\begin{aligned} {\mathbf {J}}\cdot {\mathbf {n}}|_{\text {in}}= & {} -\rho c QT_{\text {in}}, \end{aligned}$$

6b $$\begin{aligned} {\mathbf {J}}\cdot {\mathbf {n}}|_{\text {out}}= & {} \rho c(T{\mathbf {u}}\cdot {\mathbf {n}})|_{\text {out}}, \end{aligned}$$

where ${\mathbf {n}}$ is an outward pointing normal vector. We assume the diffusive transfer of heat through the walls of the calyx is proportional to the temperature difference (Newton’s law of cooling) and that the fluid cannot move through the walls, ${\mathbf {u}}\cdot {\mathbf {n}} = 0$,

7 $$\begin{aligned} {\mathbf {J}}\cdot {\mathbf {n}}|_{\text {walls}} = h(T - T_0)|_{\text {walls}}, \end{aligned}$$

where *h* is the heat transfer coefficient (with units Watts/m$^2$K) and $T_0$ is the temperature outside the walls. We assume that the calyx is initially at temperature $T_{\text {ic}}$ (likely to be body temperature, $37\,^{\circ }\text {C}$),

8 $$\begin{aligned} T({\mathbf {x}},0) = T_{\text {ic}}. \end{aligned}$$

### Integrated model

To derive an integrated model, we integrate Eq. (5) over the volume of the calyx

9 $$\begin{aligned} \iint _{\Omega }\frac{\partial {\hat{T}}}{\partial {\hat{t}}}\mathrm{d}\Omega + \iint _{\Omega }\nabla \cdot {\mathbf {J}}\,\mathrm{d}\Omega = \iint _{\Omega }\hat{{\mathcal {W}}}\delta (\hat{{\mathbf {x}}}-\hat{{\mathbf {x}}}_0)\mathrm{d}\Omega , \end{aligned}$$

Utilising the divergence theorem, we obtain

10 $$\begin{aligned} \iint _{\Omega }\frac{\partial T}{\partial t}\mathrm{d}\Omega + \oint _{\Gamma }{\mathbf {J}}\cdot {\mathbf {n}}\,\mathrm{d}\Gamma = {\mathcal {W}}(t), \end{aligned}$$

where $\Gamma = \Gamma _{\text {in}} + \Gamma _{\text {out}} + \Gamma _{\text {walls}}$. Applying boundary conditions (6a) , (6b) and (7), we obtain

11 $$\begin{aligned}&\rho c\frac{\mathrm{d}}{\mathrm{d}t}\iint _{\Omega }T\,\mathrm{d}\Omega - \rho c QT_{\text {in}} + \rho c\oint _{\Gamma _{\text {out}}}T{\mathbf {u}}\cdot {\mathbf {n}}\,\mathrm{d}\Gamma _{\text {out}}\nonumber \\&\quad + h\oint _{\Gamma _{\text {walls}}}(T-T_0)\,\mathrm{d}\Gamma _{\text {walls}} = W(t), \end{aligned}$$

where $W = V{\mathcal {W}}(t)$ is the laser power in Watts. We define the average temperature

12 $$\begin{aligned} {\bar{T}} = \frac{1}{V}\iint _{\Omega }{\hat{T}}\,\mathrm{d}\Omega , \end{aligned}$$

where *V* is the volume of the calyx and we make the ansatz

13 $$\begin{aligned} T|_{\Gamma _{\text {out}}} = T|_{\Gamma _{\text {walls}}} = {\bar{T}}. \end{aligned}$$

Under these assumptions, Eq. (11) reduces to

14 $$\begin{aligned} \rho c V\frac{\mathrm{d}{\bar{T}}}{\mathrm{d}t} -\rho c QT_{\text {in}} + \rho cQ{\bar{T}} + hs({\bar{T}}-T_0) = W(t), \end{aligned}$$

where *s* is the cross-sectional area of the calyx walls.

### Model solution (flow)

Theoretically, any function can be adopted for *W*(*t*). For a constant function $W(t) = W$, assuming $Q\ge 0$, Eq. (14) subject to (8) can be solved analytically to obtain

15 $$\begin{aligned}&{\bar{T}}(t; T_{\text {ic}}, W)\nonumber \\&\quad = T_0\left( \frac{\alpha + {\hat{T}}_{\text {in}} + \exp (-AtQ/V)\left( \alpha ({\hat{T}}_{\text {ic}}-1)+{\hat{T}}_{\text {ic}}-{\hat{T}}_{\text {in}} - {\hat{W}}\right) + {\hat{W}}}{A}\right) , \end{aligned}$$

where ${\hat{T}}_{\text {in}} = T_{\text {in}}/T_0$, ${\hat{T}}_{\text {ic}} = T_{\text {ic}}/T_0$, ${\hat{W}} = W/(\rho c T_0 Q)$, $\alpha = \beta /(Q\rho c)$, and $A = 1+\alpha$. We note we can immediately determine the steady state of Eq. (15) by taking the limit as $t\rightarrow \infty$ to obtain

16 $$\begin{aligned} {\bar{T}}^{\star } = T_0\left( \frac{\alpha + {\hat{T}}_{\text {in}} + {\hat{W}}}{A}\right) . \end{aligned}$$

For $W = 0$, we note that

17 $$\begin{aligned} {\bar{T}}^{\star } = T_0\left( \frac{{\hat{T}}_{\text {in}} + \alpha }{1+\alpha }\right) , \end{aligned}$$

and hence, for $\alpha \ll 1$, ${\bar{T}}^{\star } \approx T_{\text {in}}$. Conversely, if $\alpha \gg 1$, ${\bar{T}}^{\star } \approx T_0$. This is intuitive, as $\alpha$ dictates the relationship between heat lost due to diffusion through the walls and advection out of the vessel. If heat is primarily lost due to diffusion through the walls ($\alpha \gg 1$), the temperature will equilibrate to the ambient temperature in the bath surrounding the vessel, whereas if heat is lost mainly through advection by irrigation, the temperature will plateau at the temperature of the incoming irrigation fluid.

To emulate the experiments in "Experimental setup" section , we assume the laser is switched on at $t = t_1$ and off at $t = t_2$ and approximate this behaviour with a piecewise function

18 $$\begin{aligned} W(t) = {\left\{ \begin{array}{ll} 0,\text { for }t< t_1,\\ W,\text { for }t_1\le t\le t_2,\\ 0,\text { for }t> t_2. \end{array}\right. } \end{aligned}$$

The solution for all time, assuming a firing function of the form in Eq. (18) can be written in a piecewise manner

19 $$\begin{aligned} T_{1,2,3}(t) = {\left\{ \begin{array}{ll} T_1(t) = T(t;T_0,0),\text { for }t < t_1,\\ T_2(t) = T(t; T_1(t_1),W),\text { for }t_1\le t\le t_2,\\ T_3(t) = T(t; T_2(t_2),0),\text { for }t>t_2. \end{array}\right. } \end{aligned}$$

We also define the quantities

20 $$\begin{aligned} \Delta T = {\bar{T}}-T_{\text {ic}}, \qquad t_{\text {f}} = t_2 - t_1, \end{aligned}$$

i.e., the temperature change from the initial temperature and the laser firing time, respectively.

### Model solution (no flow)

The solution to Eq. (14) subject to (8) for $Q = 0$ (i.e., no irrigation flow) and $W(t) = W$ is

21 $$\begin{aligned} {\bar{T}}(t; T_0, W) = \frac{\beta T_0 + W -\exp (-\beta t/\rho c V)\left( \beta (T_0-T_{\text {ic}})+W\right) }{\beta }. \end{aligned}$$

The associated steady state is thus

22 $$\begin{aligned} {\bar{T}}(t; T_0, W) = T_0 + W/\beta . \end{aligned}$$

Assuming a piecewise function for *W*, Eq. (18), the solution can still the be written in the form of Eq. (19), although with the form of ${\bar{T}}$ provided by Eq. (22).

## Appendix D: Solving for $t_{\text {f}}$ such that $t_{43} = 120$ min

To solve

23 $$\begin{aligned} t_{43} = \int R^{43-T(t)}\mathrm{d}t = 120\text { min}, \end{aligned}$$

we determine an analytical expression for

24 $$\begin{aligned} \int _a^b C^{43-T(t)}\mathrm{d}t , \end{aligned}$$

where *C* is an arbitrary constant. If $T^{\star } > 43\,^{\circ }\text {C}$, we split the integral into four regions:

25 $$\begin{aligned} {\left\{ \begin{array}{ll} \text {I) } &{}T(t) \le 43\,^{\circ }\text {C}, \, t< t_{\text {f}},\\ \text {II) } &{}T(t)> 43\,^{\circ }\text {C}, \, t< t_{\text {f}},\\ \text {III) } &{}T(t)> 43\,^{\circ }\text {C}, \, t < t_{\text {f}},\\ \text {IV) } &{}T(t) \le 43\,^{\circ }\text {C}, \, t > t_{\text {f}}. \end{array}\right. } \end{aligned}$$

If $T^{\star } < 43\,^{\circ }\text {C}$, we simply consider:

26 $$\begin{aligned} {\left\{ \begin{array}{ll} \text {I) } &{}t < t_{\text {f}},\\ \text {II) } &{}t > t_{\text {f}}. \end{array}\right. } \end{aligned}$$

Finally, if $T^{\star } < 37\,^{\circ }\text {C}$, we determine

27 $$\begin{aligned} t_{\text {f}}^{\mathrm{safe}}= \infty . \end{aligned}$$

For $T^{\star } > 37\,^{\circ }\text {C}$ we use Matlab’s fsolve function to solve for $t_{\mathrm{f}}$ such that $t_{43} = 120$ minutes.
